# Defining bovine CpG epigenetic diversity by analyzing RRBS data from sperm of Montbéliarde and Holstein bulls

**DOI:** 10.3389/fcell.2025.1532711

**Published:** 2025-02-20

**Authors:** Emanuele Capra, Barbara Lazzari, Paolo Cozzi, Federica Turri, Riccardo Negrini, Paolo Ajmone-Marsan, Alessandra Stella

**Affiliations:** ^1^ Institute of Agricultural Biology and Biotechnology, National Research Council IBBA CNR, Lodi, Italy; ^2^ Department of Animal Science, Food and Nutrition – DIANA, and Romea and Enrica Invernizzi Research Center on Sustainable Dairy Production - CREI, Università Cattolica del Sacro Cuore, Piacenza, Italy

**Keywords:** epigenetic, cattle, breed, sperm, DNA methylation

## Abstract

**Introduction:**

Breed epigenetic diversity was recently detected in pig muscle and cattle blood, probably as a result of long-term selection for morphological adaptive and quantitative traits, persisting after embryo epigenetic reprogramming.

**Methods:**

In our study, breed epigenetic diversity in the male germline from Holstein (H) and Montbéliarde (M) bulls was investigated using Reduced Representation Bisulfite Sequencing (RRBS) data publicly available at the NCBI database. Open-source Whole Genome Sequencing (WGS) data from H and M animals were used to estimate genetic diversity between the two breeds and, thus, correctly assess CpG positions with low frequencies or absence of SNPs.

**Results:**

Sperm epigenetic diversity was studied in 356,635 SNP-free CpG positions, and a total of 6,074 differentially methylated cytosines (DMCs) were identified. The analysis of the DMCs pattern of distribution revealed that DMCs: i) are partially associated with genetic variation, ii) are consistent with epigenetic diversity previously observed in bovine blood, iii) present long-CpG stretches in specific genomic regions, and iv) are enriched in specific repeat elements, such as ERV-LTR transposable elements, ribosomal 5S rRNA, BTSAT4 Satellites and long interspersed nuclear elements (LINE).

**Discussion:**

This study, based on publicly available data from two cattle breeds, contributes to the identification and definition of distinct epigenetic signatures in sperm, that may have potential implications for mammalian embryo development.

## Introduction

Epigenome variation in livestock has been explored for its influence on production (e.g., milk production), disease traits, reproduction, and environmental adaptation (Schenkel, 2021). Studies in insects, fishes and birds have already reported how different epigenetic mechanisms can contribute to phenotypic variability in response to environmental adaptation (Alvarado et al., 2015; Fanti et al., 2017; Gore et al., 2018; Heckwolf et al., 2020; Lindner et al., 2021). Epigenetic inheritance is only minimally documented in mammals, with few well-characterized examples. One classic case is the agouti locus, where the CpG methylation level in repetitive elements of the Class II endogenous retrovirus (ERV) family in the intracisternal A-particle (IAP) insertions, upstream of the agouti gene, is epigenetically inherited and influences gene expression and mouse coat color (Morgan et al., 1999). However, the driving mechanisms governing epigenetic inheritance in mammals remain poorly understood due to the extensive genome-wide epigenetic reprogramming that takes place during mammalian development (Zeng and Chen, 2019). In the preimplantation embryos and in the germline a global epigenetic reprogramming occurs, giving rise to changes in DNA methylation. During fertilization, parental epigenomes undergo a remarkable demethylation in the zygote, followed by remethylation during the subsequent cell division, necessary to differentiate and direct cells toward their future lineages and ensure that cell type specification is a one-way street. However, the same epigenetic somatic signatures need to be erased in the primordial germ cells (PGCs) by a comprehensive reprogramming process, able to redirect DNA methylation for the establishment of sex-specific and germ cell-specific epigenetic signatures through a specialized processes of meiotic maturation and fertilization (Messerschmidt et al., 2014).

The genome-wide methylation reprogramming driven by demethylation that is observed in mammalian embryos in early preimplantation development, is conserved in many species (Dean et al., 2001). Remethylation typically occurs from the eight-cell stage, but with different rates in different mammalian species. In cattle, a considerable *de novo* methylation is observed in embryos between the eight-cell and the 16-cell steps (Dean et al., 2001; Triantaphyllopoulos et al., 2016). Nonetheless, specific genomic regions resist the global demethylation and are able to escape from global epigenetic reprogramming (Jiang et al., 2018).

It was reported that muscle CpG methylation shows important differences across breeds in many genes, probably as a result of long-term selection for quantitative traits (Ponsuksili et al., 2019). Breed epigenetic diversity could be explained by genetic variation as a consequence of the selection process. However, we recently evaluated inter-species epigenetic diversity in *Bos taurus* and *Bos indicus* providing results that support the assumption that epigenetic diversity is partially independent from genotype and may potentially impact on anatomical morphogenesis and breed traits (Capra et al., 2023). This assumption requires that the epigenetic signature is conserved in various tissues including the male and female germlines, to be transferred through generations. The abovementioned studies reported breed epigenetic differences in two somatic tissues: muscle (Ponsuksili et al., 2019) and blood (Capra et al., 2023). We decided to investigate breed epigenetic diversity in the male germline by evaluating differentially methylated CpGs in two different cattle breeds by using publicly available Reduced Representation Bisulfite Sequencing (RRBS) data collected from sperm of Montbéliarde and Holstein bulls (Costes et al., 2022; Johnson et al., 2022). Following this approach, we were able to identify epigenomic elements characterizing bovine inter-breed epigenetic diversity.

## Materials and methods

### NCBI bioproject and biosamples selection

This study utilized publicly available data for assessing sperm epigenetic and genetic variation in different cattle breeds. Fastq sequences were selected from the NCBI Bioproject database and retrieved from NCBI SRA. To ensure that comparisons between breeds were based on a substantial number of animals having an adequate sequencing coverage (granting to have a high number of cytosines with at least 10X coverage), results were filtered taking into account the following criteria: i) library preparation method (RRBS) ii) high number of available samples (n > 8), iii) sequencing coverage (RRBS>20 million reads). Two projects met the above-mentioned criteria and were deemed comparable: PRJEB46371 for Montbéliarde breeds (Costes et al., 2022), and bioprojects PRJEB35854 for Holstein (Johnson et al., 2022). Bioproject PRJEB35854 contains a total of 18 Biosamples from 9 Holstein bulls collected at two different timepoints (55-59 and 69–71 weeks). Bioproject PRJEB46371 contains a total of 120 Biosamples from 120 Montbéliarde bulls collected at different ages. Nine samples from the Holstein breed (69–71 weeks) and other nine randomly chosen samples with comparable age (74–82 weeks) for the Montbéliarde breed were selected for this study (Supplementary Table S1). The chosen age-window of 69–82 weeks (approximately 16–19 months) aligns well with the period of full sexual maturity for both breeds (Barth and Waldner, 2002; Rawlings et al., 2008). Finally, to assess genetic variation in the two breeds, NCBI publicly available WGS data form Holstein and Montbéliarde (Bioproject PRJEB9343) were used (Boussaha et al., 2015). Finally, another RRBS dataset (Bioproject PRJNA433629) with data from cells isolated in different stages of cattle embryogenesis: 2-Cell (2C), Compact Morula (CM), Blastocyst (B) and Spermatozoa (S), from Jiang et al., 2018, was included in this study to evaluate if breed specific CpG methylation variation can potentially escape from epigenetic reprogramming.

### Bioinformatic analysis

Public RRBS raw fastq sequences were downloaded from the NCBI SRA database and analysed via nf-core (Garrison and Marth, 2012), using the nf-core/methylseq v1.5 pipeline (doi: 10.5281/zenodo.2555454). Bismark v0.22.3 (https://www.bioinformatics.babraham.ac.uk/projects/bismark/) was used as aligner. FastQC v0.11.9 (http://www.bioinfor.matics.babraham.ac.uk/projects/fastqc/). is included in the pipeline for raw data quality control, Trim Galore v0.6.4 (http://www.bioinformatics.babraham.ac.uk/projects/trim_galore/) performs adapter sequence trimming, and Qualimap v2.2.2 (Ewels et al., 2020), is used for alignment quality control. As reference genome, *B. taurus* ARS-UCD1.2 (GCF_002263795.1) was chosen, with the addition of chromosome Y from Btau_5.0.1 (NC_016145.1). Methylation calls were extracted with Bismark methylation extractor v0.22.3.

WGS raw fastq sequences were also downloaded from the NCBI SRA database and analyzed with a custom pipeline built on the nf-core infrastructure. As for RRBS, the pipeline includes FastQC and TrimGalore, sequences were then aligned to the same reference genome with BWA-MEM v0.7.17, and SNP detection was performed with FreeBayes v1.3.6. Accessory steps were performed with bcftools v1.16, Picard v2. 27.4, samtools v1.16.1, and Tabix v1.12. For the variance partition analysis, the cytosines of interest were extracted from the whole dataset in pileup format using samtools pileup, and analyzed with the ‘variancePartition’ R package (http://bioconductor.org/packages/release/bioc/html/variancePartition.html).

In order to estimate breed genetic diversity, any SNP type with an allele frequency of more than 10% was considered. The Seqmonk software v1.47.1 (https://github.com/s-andrews/SeqMonk), was chosen for visualization and analysis of the Bismark output. To identify cytosines suitable for differential methylation analysis discarding polymorphic positions between the two breeds, cytosines matching SNPs in either species were removed from the analysis. Differentially methylated cytosines (DMCs) between the two breeds were detected among cytosines with at least 10X coverage in all nine animals, using logistic regression (False Discovery Rate (FDR) ≤ 0.05). Random subsets from the identified R-Cs depurated from DMCs were created using the “sample_n” function in R, t-test analysis was used to evaluate the hypothesis that elements characterizing bovine interbreed epigenetic diversity are not exclusively due to chance (p-value <0.05 was considered significant). Gene ontology (GO) classification was performed with the Cytoscape plug-in ClueGO (Bindea et al., 2009), which integrates GO and enhances biological interpretation of large lists of genes.

## Results

### Study workflow

**FIGURE 1:**
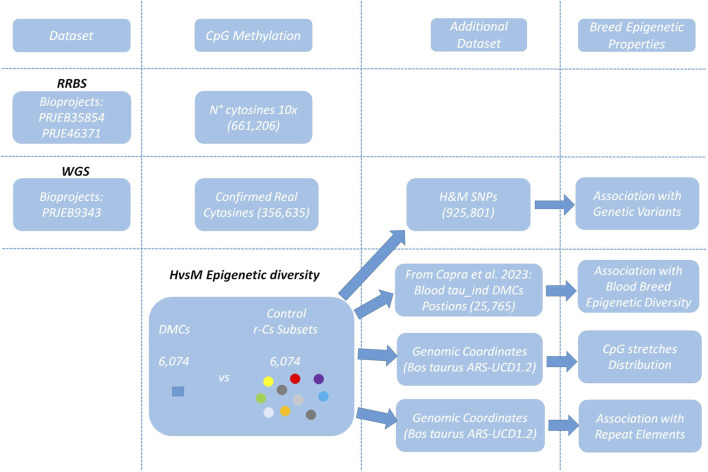
Study Workflow. Datasets available at the NCBI database are PRJEB35854 and PRJEB46371 to assess CpG methylation and PRJEB9343 for SNP identification. The differentially Methylated Cytosines dataset (DMCs) and 10 random cytosine r-Cs subsets, each containing the same number of cytosines as the DMCs dataset, were compared to other previously defined datasets to identify specific features characterizing H and M breed epigenetic diversity.

This study utilized public RRBS raw fastq sequences from Bioprojects PRJEB35854 and PRJEB46371 to assess sperm epigenetic diversity between H and M breeds. To distinguish with confidence between genomic thymines and unmethylated cytosines (which are read as thymines in bisulfite sequencing), we also included the Bioproject PRJEB9343 dataset containing whole genome sequencing (WGS) raw fastq sequences from animals from same breeds (Boussaha et al., 2015). This dataset allowed also the identification of genetic variation between the two breeds, which was superposed to the methylated cytosines dataset to remove positions overlapping SNPs. The remaining CpGs were used to find breed specific differentially methylated cytosines (DMCs). In order to thoroughly investigate breed epigenetic diversity, we generated 10 sets of randomly selected (excluding DMCs) control cytosines (r-Cs), which were used to validate the estimation of the occurrence of epigenetic variation in proximity of genomic positions associated to animal genetic variation, cattle subspecies blood epigenetic diversity, and repetitive elements. Figure 1 displays the study workflow.

### Global mapping of DNA methylation and principal component analysis (PCA) (is breed epigenetic diversity observable in sperm?)

Epigenetic variation in bull sperm from two different breeds, Holstein (H) and Montbéliarde (M), was observed by analyzing NCBI datasets from 9 animals of each breed (Bioprojects; PRJEB35854 and PRJEB46371) (Costes et al., 2022; Johnson et al., 2022).

RRBS reads were mapped to the *B. taurus* genome (ARS-UCD1.2, plus chromosome Y from Btau_5.0.1). In RRBS analysis, a cutoff of 10X coverage was imposed. The average number of reads per sample was 32.2 M (range: 25.5M– 40.7 M) (Supplementary Table S1). Within the CpG enriched regions represented in RRBS, 30.2% were methylated. The dataset counts 5,182,326 cytosines within the CpG context with 10X coverage per position in at least one sample. To be sure to focus our attention on cytosines well represented in all the animals under investigation, we extracted from this dataset 661,206 CpGs with at least 10X coverage in each of the aligned samples.

**FIGURE 2:**
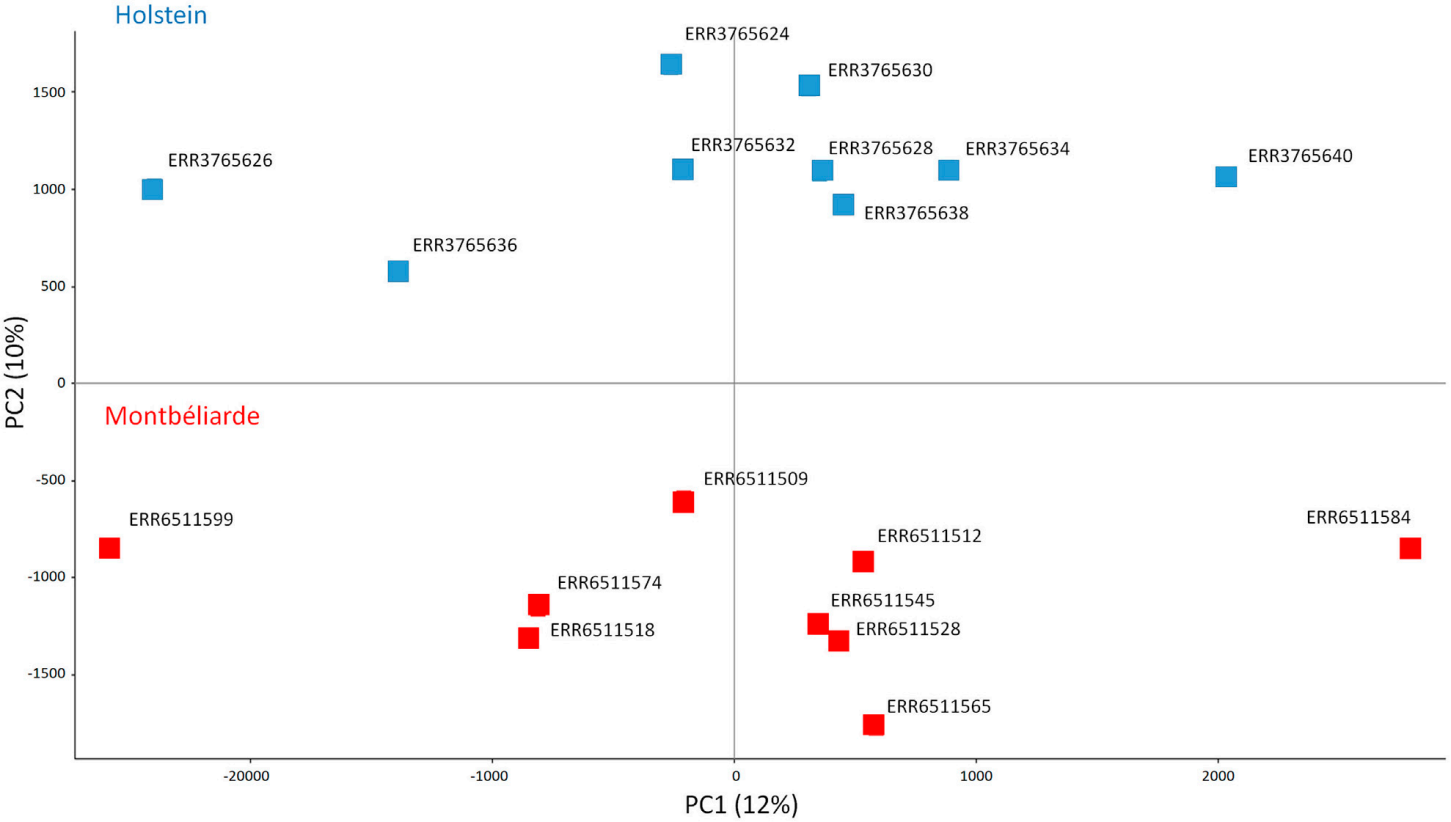
PCA of bull sperm CpG methylation considering the selected 356,635 cytosines. In blue Holstein (n = 9), in red Montbéliarde (n = 9).

Due to bisulfite treatment, differential CpG methylation calling may be affected by sequence polymorphism with respect to the genotype of the reference sequence used in the alignment procedures of both the RRBS and WGS pipelines. Unfortunately, no public WGS sequence of the same animals selected for RRBS analysis was available, thus, we analyzed a subset of NCBI publicly available WGS data from different animals of the same breeds (M and H, Bioproject PRJEB9343) (Boussaha et al., 2015) (Supplementary Table S2), to detect breed polymorphisms and clean CpG positions identified by RRBS analysis, retaining exclusively those positions not overlapping polymorphisms observed in either the M and H breeds. At the end of this procedure, a total of 356,635 CpG positions were selected, representing a clean and reliable dataset to be used as input for all the subsequent analyses on methylation distribution. A principal component analysis (PCA) of the samples according to the CpG methylation level of the selected 356,635 cytosines well separates sperm from H and M animals along PC2 (Figure 2).

### Differentially methylated cytosines (DMCs) and genetic variation (are genetics and epigenetics overlapping but distinguishable?)

A total of 6,074 DMCs were identified between the H and M breeds (Supplementary Table S3). These DMCs are differently distributed along chromosomes (Supplementary Figure S1). Interestingly, the Y chromosome shows the highest number of DMCs, whereas on the X chromosome only a few regions exhibit changes in DNA methylation between two breeds.

**FIGURE 3:**
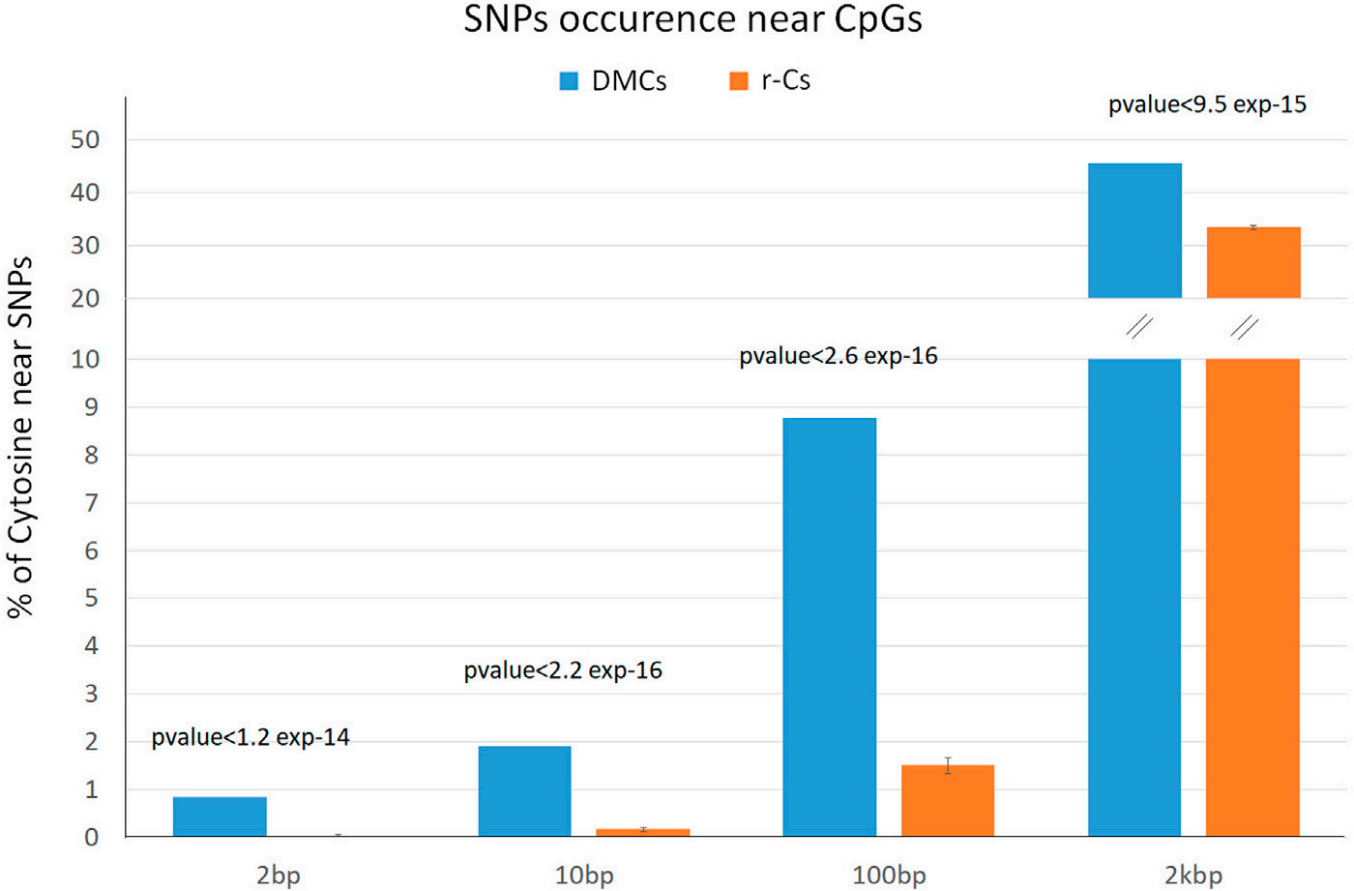
Percentage of differentially methylated cytosines (DMCs, blue) and random cytosines (r-Cs, orange) near SNPs between H and M breeds in the four considered intervals (2 bps, 10 bps, 100 bps, and 2 kbs from a SNPs position).

DMCs distribution was compared with that of 10 control subsets of random Cytosines (r-Cs, n = 6,074 for each of the 10 datasets, which do not include DMCs) to highlight possible patterns of distribution of differential methylation among breeds not occurring by chance. Because epigenetic and genetic diversity are partially correlated, we tested if DMCs have a preferential distribution close to SNP positions when compared to r-Cs. H and M genetic diversity was assessed from WGS analysis (Bioproject PRJEB9343) (Boussaha et al., 2015), which identified a total of 925,801 SNPs along the reference genome. To validate the use of the 10 r-Cs subsets in our analyses, we initially tested whether they were representative of the genome-wide distribution of r-Cs. The incidence of SNPs was calculated for the whole 350,561 r-Cs covered by the RRBS analysis (i.e., the 356,635 CpG positions selected after removal of the positions matching breed polymorphisms minus the 6,074 DMCs), showing consistency with the percentage found in the 10 random subsets (Supplementary Table S4). By comparing distances of DMCs and of the 10 r-Cs subsets from SNP positions and determining their statistical significance by running a t-test analysis, we observed that the former fall near SNPs with a higher frequency with respect to the latter (which represent the distribution expected by chance). To represent this situation, we collected DMCs and r-Cs in four groups according to their genomic distance from breed SNPs (up to 2 bps, up to 10 bp, up to 100 bps, and up to 2 kbs from a SNPs position; Figure 3; Supplementary Table S4). In each of the considered intervals, we observe a significantly higher proportion of DMCs rather than r-Cs that fall close to SNPs. If we consider the 100 bps interval, the proportion of cytosines close to SNPs is 8.78% DMCs vs. 1.50% r-Cs, while this difference lowers in the 2 kbs interval, when drawing away from SNPs. This distribution suggests that many epigenetic differences could be driven by genetic variation and *vice versa*.

A functional analysis of breed epigenetic diversity was run on a dataset comprising 533 DMCs representing positions where breeds exhibit their most evident differences. The dataset includes only DMCs showing the highest methylation variation (more than 20%) between H and M, and excludes DMCs falling less or equal than 100 bps from SNPs, that could be related to genetic variation. DMCs selected according to these criteria are enriched in genes involved in regulation of Wnt signaling pathway, GTPase activity signal transduction, cell communication, multicellular organism development, system development and cell morphogenesis (Supplementary Table S5). We finally evaluated if breed epigenetic signatures can be maintained during epigenetic reprogramming. We explored data from cells isolated in different stages of cattle embryogenesis: 2C, CM, B and S from the Bioproject PRJNA433629. Due to the low CpG coverage of the PRJNA433629 dataset, the methylation percentage for different embryonic developmental stages was calculated for each cellular type by combining replicates, and the resulting datasets were compared to DMCs obtained by H and M sperm methylation comparison. Only a little proportion of the DMCs individuated by our analysis (308/6,074) showed at least a CpG 5X coverage in all the four developmental stages of the PRJNA433629 dataset. Hierarchical clustering showed that methylation profile of the PRJNA433629 sperm sample was similar to those from H and M sperm, and clustered more similar to blastocyst rather than 2-cell and compact morula stages. Some cytosines were consistent with the occurrence of a demethylation and re-methylaion reprogramming wave (Supplementary Figure S2). These results, however, should be interpreted with caution because of the low level of CpG coverage in the PRJNA433629 dataset, and because only a portion of the entire epigenome is represented in RRBS experiments. In fact, only a few genes (LOC112442278, LOC112446467, PPM1B, LOC112443250 and RN18S1) are present in the genomic regions interested by these DMCs, which for the overmentioned technical reasons represent only a very little subset of the DMCs potentially involved in the methylation reprogramming wave.

### Differentially methylated cytosines (DMCs) and comparison with subspecies blood epigenetic diversity. (is epigenetic breed diversity conserved in different tissues?)

**FIGURE 4:**
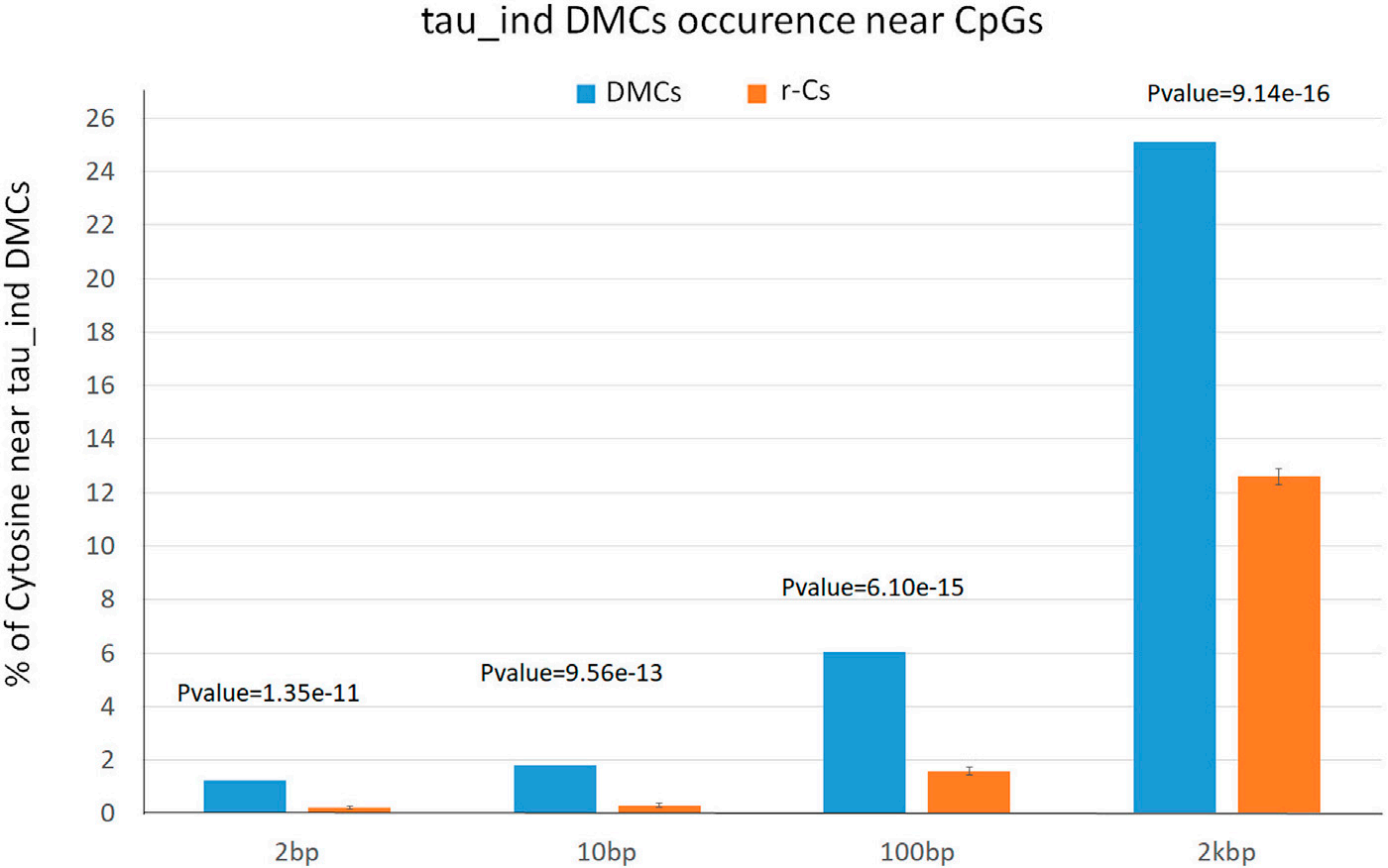
Percentage of differentially methylated cytosines (DMCs, blue) and SNP free cytosines (r-Cs, orange) near subspecies-specific differentially methylated cytosines (DMCs between *Bos taurus* and *Bos indicus*: 2 bps, 10 bps, 100 bps, 2 kbs from a SNPs position).

We recently assessed *Bos* subspecies epigenetic diversity in blood from Angus and Nellore identifying 25,765 DMCs between *B. taurus* and *B. indicus* (tau_ind_DMCs) (Capra et al., 2023). In order to assess if blood bovine subspecies epigenetic diversity can be associated to breed epigenetic diversity in sperm, we evaluated whether H vs. M DMCs were enriched for tau_ind_DMCs with respect to r-Cs control subsets. Comparison of the H vs. M DMCs (n = 6,074) with those found in this previous study showed that 24.0%, 5.5%, 1,7%, and 1.2% cytosines are close or adjacent (within 2,000 bps, 100 bps, 10 bps, 2 bps) to tau_ind_DMCs. On the contrary, the average number of cytosines belonging to the 10 control subsets of that are close or adjacent to tau_ind_DMCs, was statistically lower (13.87%, 1.59%, 0.31%, and 0.22%) for less than 2000 bps, 100 bps, 10 bps and 2 bps intervals, respectively, as confirmed by t-test analysis (Figure 4; Supplementary Table S6). This significant difference highlights a partially overlapping pattern of genomic localization of breed epigenetic variation in blood and sperm.

### Differentially methylated cytosines (DMCs) organization. (do epigenetic breed diversity variations show specific features?)

**FIGURE 5:**
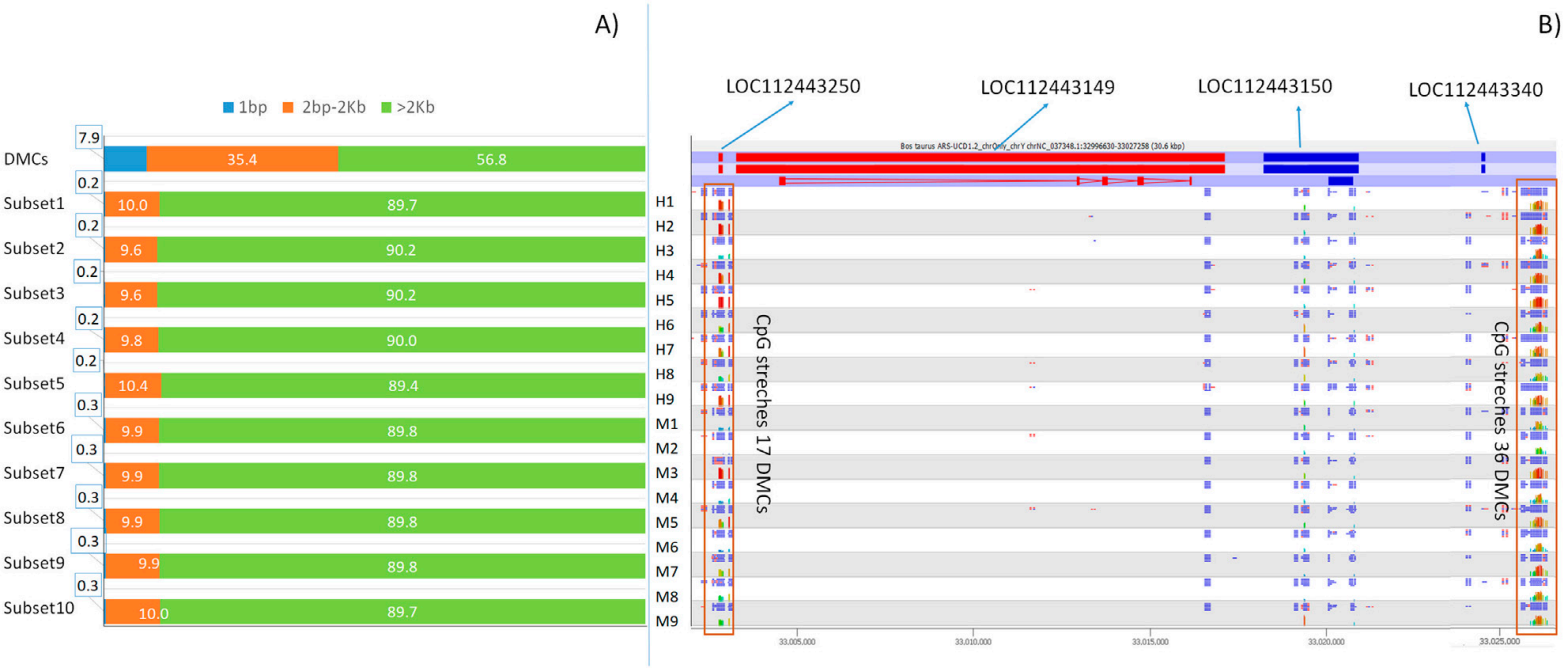
**(A)** Percentage of CpGs according to the proximity to the next CpG (1 bp, between 2 bps and 2 kbs and more than 2 kbs) for DMCs and the ten random r-Cs subsets. **(B)** The *Bos taurus* ARS-UCD1.2 NC_037348.1:32996630-3027258 region and the level of methylation in 9 H and 9 M animals for DMC stretches (DMCs distance from the adjacent cytosine below 2 kbs) encompassing LOC112443250 and LOC112443340 5S rRNA genes, which surround LOC112443149 and LOC112443150, codifying liprin-alpha-1-like and S-antigen like proteins.

A previous study showed that *taurus* and *indicus* subspecies present a high number of adjacent differentially methylated CpGs (DMC stretches) (Capra et al., 2023). Here, H and M show a higher percentage of CpG stretches with at least 2 consequent cytosines at a distance of 2,000 bp or less in DMCs, when compared to r-Cs subsets (Figure 5A). Moreover, the number of adjacent cytosines (i.e., cytosines included in stretches) occurring by chance in r-Cs is much lower with respect to the one observed in DMCs (p-value <2.2e-22) (Figure 5A; Supplementary Table S7). Considering that we are analyzinng RRBS experiments, we should assume that the sequenced cytosines are mostly contained in methylated regions. Nonetheless, we observe these differences in the distribution within CpG stretches of DMCs versus other cytosines that are methylated but not differentially methylated between H and M. Interestingly, among genes having longest CpG stretches, LOC112443340 (36 adjacent DMCs in 381bps) and LOC112443250 (16 adjacent DMCs in 304 bps), both coding for 5S ribosomal RNA, present a specific feature by sorrounding a 25 Kb genomic tract encompassing other two genes (LOC112443149 and LOC112443150, Figure 5B).

### Differentially methylated cytosines (DMCs) and repeat regions. (do breed epigenetic variations preferentially overlap with regions containing repeats?)

**FIGURE 6:**
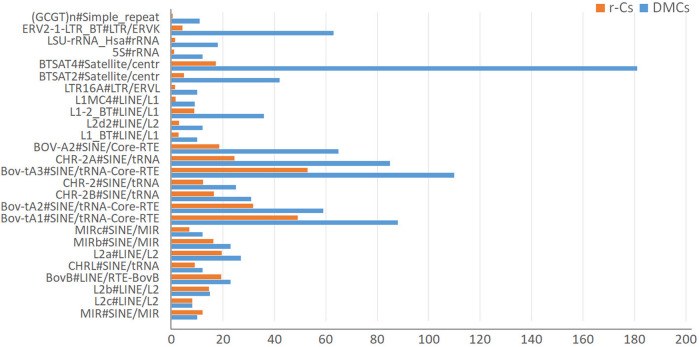
Plot of the number of CpGs overlapping repeats for differentially methylated cytosines (DMCs) and control subsets of random SNP-free cytosines (r-Cs).

We finally tested if DMCs are preferentially distributed within repeated regions. In general, in our datasets DMCs are more abundantly located in repetitive regions than due to chance (i.e., than in r-Cs), mostly occurring in ERV-LTR, 5S rRNA, BTSAT4 Satellites repeat elements and long interspersed nuclear elements (LINE) (Figure 6; Supplementary Table S8).

DMCs overlapping positions show enrichments and depletions for specific classes of repeat elements. In particular, when compared to control subsets, DMCs show enrichment in specific classes of ERV-LTR, 5S rRNA, BTSAT4 Satellites repeat elements and long interspersed nuclear elements (LINE).

## Discussion

Using publicly available RRBS data, our work characterized interbreed epigenetic diversity in the cattle male germline. Since 1988, The National Center for Biotechnology Information (NCBI) collects open-access nucleotide and genomic sequences from many organisms, including next-generation sequencing data from genomic, transcriptomic and epigenetic studies (Kodama et al., 2012; NCBI Resource Coordinators, 2013), contained in the sequence read archive (SRA). SRA data are organized through BioProject and BioSample records that link to corresponding data stored in archival repositories, thus facilitating and improving the organization of the metadata describing the experiment (Barrett et al., 2012). In order to study cytosines free of breeds polymorphisms, thus avoiding biases due to incorrect CpG methylation calls, we identified two suitable RRBS experiments (Costes et al., 2022; Johnson et al., 2022) and a WGS dataset for the same two breeds (Boussaha et al., 2015), that were processed following previously optimized strategies (Capra et al., 2023).

We observed epigenetic specificity characterizing cattle breed diversity. First of all, epigenetic variation in H and M sperm in part adheres to the pattern of genomic regions showing CpG methylation variation in blood from *B. taurus* and *B. indicus* subspecies (Capra et al., 2023). Secondly, sperm epigenetic diversity partially correlates with genetic differences in the two breeds (Heyn et al., 2013; Zhi et al., 2013). Another aspect is that breed epigenetic diversity exhibits a high level of coordination in methylation, represented by the presence of adjacent CpG sites that are differentially co-methylated (methylated CpG stretches). A similar CpG methylation configuration was also previously observed in different cell types (Shoemaker et al., 2010) and tissues (Guo et al., 2017).Finally, we found that breed epigenetic signature co-localizes with different classes of repetitive elements, indicating that repeat elements play an important and conserved role in the establishment of inter-breed epigenetic variability. DMCs were enriched in Endogenous Retrovirus 2 with long terminal repeats ERV2 LTRs, 5S rRNA, BTSAT4 Bovine satellite and LINEs. Interestingly, both ERV LTRs repeat and ribosomal DNA (rDNA) act as important transcriptional regulatory elements with silencing or activation of the expression of different sets of genes in mammals, which is mediated by DNA methylation (Thompson et al., 2016; Tchurikov and Kravatsky, 2021; Zeng et al., 2022). We can consider the well-known example of mammalian epigenetic transmission mediated by repetitive elements of the Class II endogenous retrovirus (ERV) intracisternal A-particle (IAP), which methylation influences the expression of the Agouti coat colour gene locus in mouse (Blewitt et al., 2006; Bertozzi and Ferguson-Smith, 2020). A number of studies suggest transgenerational epigenetic inheritance (TEI) of DNA methylation changes, which involves genomic regions that are thought to almost partially escape epigenetic reprogramming through generations (Xavier et al., 2019). As recently observed in sheep (Braz et al., 2023), a consistent part of these methylation signatures falls within LINEs. H and M epigenetic comparison also evidenced a variation of CpG methylation in two adjacent 5SrRNA regions surrounding a 25 Kb genomic region containing the genes codifying a liprin-alpha-1-like (known to be important for axon guidance and mammary gland development) and S-antigen like proteins. Interestingly, the same 5SrRNA region has been observed to be differentially methylated between mammary gland tissues from Holstein cows producing milk with high and low protein contents (Wang et al., 2021). We cannot exclude that part of the sperm CpG methylation variability in the two breeds could be in part associated to semen quality in H and M animals. In fact, methylation within BTSAT4 was also previously observed to change in high and low motile *B. taurus* sperm populations (Capra et al., 2019). However, most of the epigenetic variation between the two breeds was observed to target genes related to cell signaling, multicellular organism development, system development and cell morphogenesis. Interestingly, H and M breed epigenetic diversity deeply affects Wnt/β-catenin signaling, an essential regulator in embryonic development and adult tissue homeostasis (Liu et al., 2022). Several experiments provide emerging evidence that sperm epigenome serves as a model for embryonic development (Lismer and Kimmins, 2023). Human and animal exposure to different environmental factors was linked to sperm methylation alteration, targeting important reproductive and developmental loci associated with offspring phenotypes (Rakyan et al., 2003; Lismer et al., 2024). Our data show that HvsM DMCs could maintain the methylation state after epigenetic reprogramming, particularly in long-CpG stretches close to genes that have a potential impact on anatomical morphogenesis and breed traits (LOC112442278, LOC112446467, PPM1B, LOC112443250, RN18S1; Wang et al., 2021; Capra et al., 2023). On the other hand, more in depth studies are necessary to fully understand the impact of breed epigenetic diversity on embryo development and to fully explore the rules governing breed epigenetic inheritance.

## Conclusion

In conclusion, interbreed epigenetic diversity assessed by RRBS sperm profiling in H and M animals showed specific CpG methylation signatures, with peculiar characteristics. H and M CpG methylation variation partially correlates with blood bovine interspecies methyl CpG diversity, with H and M genetic diversity, it presents many adjacent co-methylated CpG sites and is enriched in specific repeat elements. Many important questions remain unanswered by our study. Further studies will be necessary to fully trace the existence of a breed epigenetic imprint shared among different tissues and to understand how breed epigenetic signatures can persist after embryo epigenetic reprogramming and possibly contribute to breed differentiation.

## Data Availability

Publicly available datasets were analyzed in this study. This data can be found here: (https://www.ncbi.nlm.nih.gov/bioproject/?term=PRJEB46371, https://www.ncbi.nlm.nih.gov/bioproject/?term=PRJEB35854, https://www.ncbi.nlm.nih.gov/bioproject/?term=PRJEB9343).

## References

[B1] Alvarado S. Rajakumar R. Abouheif E. Szyf M. (2015). Epigenetic variation in the Egfr gene generates quantitative variation in a complex trait in ants. Nat. Commun. 6, 6513. 10.1038/ncomms7513 25758336

[B2] Barrett T. Clark K. Gevorgyan R. Gorelenkov V. Gribov E. Karsch-Mizrachi I. (2012). BioProject and BioSample databases at NCBI: facilitating capture and organization of metadata. Nucleic Acids Res. 40, D57–D63. 10.1093/nar/gkr1163 22139929 PMC3245069

[B3] Barth A. D. Waldner C. L. (2002). Factors affecting breeding soundness classification of beef bulls examined at the Western College of Veterinary Medicine. Can. Vet. J. 43, 274–284. 11963661 PMC339235

[B4] Bertozzi T. M. Ferguson-Smith A. C. (2020). Metastable epialleles and their contribution to epigenetic inheritance in mammals. Semin. Cell Dev. Biol. 97, 93–105. 10.1016/j.semcdb.2019.08.002 31551132

[B5] Bindea G. Mlecnik B. Hackl H. Charoentong P. Tosolini M. Kirilovsky A. (2009). ClueGO: a Cytoscape plug-in to decipher functionally grouped gene ontology and pathway annotation networks. Bioinformatics 25, 1091–1093. 10.1093/bioinformatics/btp101 19237447 PMC2666812

[B6] Blewitt M. E. Vickaryous N. K. Paldi A. Koseki H. Whitelaw E. (2006). Dynamic reprogramming of DNA methylation at an epigenetically sensitive allele in mice. PLoS Genet. 2, e49. 10.1371/journal.pgen.0020049 16604157 PMC1428789

[B7] Boussaha M. Esquerré D. Barbieri J. Djari A. Pinton A. Letaief R. (2015). Genome-wide study of structural variants in bovine Holstein, Montbéliarde and normande Dairy breeds. PLoS One 10, e0135931. 10.1371/journal.pone.0135931 26317361 PMC4552564

[B8] Braz C. U. Passamonti M. M. Khatib H. (2023). Characterization of genomic regions escaping epigenetic reprogramming in sheep. Environ. Epigenetics 10, dvad010. 10.1093/eep/dvad010 PMC10944287 38496251

[B9] Capra E. Lazzari B. Milanesi M. Nogueira G. P. Garcia J. F. Utsunomiya Y. T. (2023). Comparison between indicine and taurine cattle DNA methylation reveals epigenetic variation associated to differences in morphological adaptive traits. Epigenetics 18, 2163363. 10.1080/15592294.2022.2163363 36600398 PMC9980582

[B10] Capra E. Lazzari B. Turri F. Cremonesi P. Portela A. M. R. Ajmone-Marsan P. (2019). Epigenetic analysis of high and low motile sperm populations reveals methylation variation in satellite regions within the pericentromeric position and in genes functionally related to sperm DNA organization and maintenance in *Bos taurus* . BMC Genomics 20, 940. 10.1186/s12864-019-6317-6 31810461 PMC6898967

[B11] Costes V. Chaulot-Talmon A. Sellem E. Perrier J. P. Aubert-Frambourg A. Jouneau L. (2022). Predicting male fertility from the sperm methylome: application to 120 bulls with hundreds of artificial insemination records. Clin. Epigenetics 14, 54. 10.1186/s13148-022-01275-x 35477426 PMC9047354

[B12] Dean W. Santos F. Stojkovic M. Zakhartchenko V. Walter J. Wolf E. (2001). Conservation of methylation reprogramming in mammalian development: aberrant reprogramming in cloned embryos. Proc. Natl. Acad. Sci. U. S. A. 98, 13734–13738. 10.1073/pnas.241522698 11717434 PMC61110

[B13] Ewels P. A. Peltzer A. Fillinger S. Patel H. Alneberg J. Wilm A. (2020). The nf-core framework for community-curated bioinformatics pipelines. Nat. Biotechnol. 38, 276–278. 10.1038/s41587-020-0439-x 32055031

[B14] Fanti L. Piacentini L. Cappucci U. Casale A. M. Pimpinelli S. (2017). Canalization by selection of *de novo* induced mutations. Genetics 206, 1995–2006. 10.1534/genetics.117.201079 28576865 PMC5560803

[B15] Garrison E. Marth G. (2012). Haplotype-based variant detection from short-read sequencing. arXiv Prepr. arXiv:1207.3907 [q-bio.GN]. 10.48550/arXiv.1207.3907

[B16] Gore A. V. Tomins K. A. Iben J. Ma L. Castranova D. Davis A. E. (2018). An epigenetic mechanism for cavefish eye degeneration. Nat. Ecol. Evol. 2, 1155–1160. 10.1038/s41559-018-0569-4 29807993 PMC6023768

[B17] Guo S. Diep D. Plongthongkum N. Fung H. L. Zhang K. Zhang K. (2017). Identification of methylation haplotype blocks aids in deconvolution of heterogeneous tissue samples and tumor tissue-of-origin mapping from plasma DNA. Nat. Genet. 49, 635–642. 10.1038/ng.3805 28263317 PMC5374016

[B18] Heckwolf M. J. Meyer B. S. Häsler R. Höppner M. P. Eizaguirre C. Reusch T. B. H. (2020). Two different epigenetic information channels in wild three spined sticklebacks are involved in salinity adaptation. Sci. Adv. 6, eaaz1138. 10.1126/sciadv.aaz1138 32219167 PMC7083608

[B19] Heyn H. Moran S. Hernando-Herraez I. Sayols S. Gomez A. Sandoval J. (2013). DNA methylation contributes to natural human variation. Genome Res. 23, 1363–1372. 10.1101/gr.154187.112 23908385 PMC3759714

[B20] Jiang Z. Lin J. Dong H. Zheng X. Marjani S. L. Duan J. (2018). DNA methylomes of bovine gametes and *in vivo* produced preimplantation embryos. Biol. Reprod. 99, 949–959. 10.1093/biolre/ioy138 29912291 PMC6297316

[B21] Johnson C. Kiefer H. Chaulot-Talmon A. Dance A. Sellem E. Jouneau L. (2022). Prepubertal nutritional modulation in the bull and its impact on sperm DNA methylation. Cell Tissue Res. 389, 587–601. 10.1007/s00441-022-03659-0 35779136

[B22] Kodama Y. Shumway M. Leinonen R. International Nucleotide Sequence Database Collaboration (2012). The Sequence Read Archive: explosive growth of sequencing data. Nucleic Acids Res. 40, D54–D56. 10.1093/nar/gkr854 22009675 PMC3245110

[B23] Lindner M. Laine V. N. Verhagen I. Viitaniemi H. M. Visser M. E. van Oers K. (2021). Rapid changes in DNA methylation associated with the initiation of reproduction in a small songbird. Mol. Ecol. 30, 3645–3659. 10.1111/mec.15803 33453134 PMC8359384

[B24] Lismer A. Kimmins S. (2023). Emerging evidence that the mammalian sperm epigenome serves as a template for embryo development. Nat. Commun. 14, 2142. 10.1038/s41467-023-37820-2 37059740 PMC10104880

[B25] Lismer A. Shao X. Dumargne M. C. Lafleur C. Lambrot R. Chan D. (2024). The association between long-term DDT or DDE exposures and an altered sperm epigenome-a cross-sectional study of Greenlandic inuit and South African VhaVenda men. Environ. Health Perspect. 132, 17008. 10.1289/EHP12013 38294233 PMC10829569

[B26] Liu J. Xiao Q. Xiao J. Niu C. Li Y. Zhang X. (2022). Wnt/β-catenin signalling: function, biological mechanisms, and therapeutic opportunities. Signal Transduct. Target Ther. 7, 3. 10.1038/s41392-021-00762-6 34980884 PMC8724284

[B27] Messerschmidt D. M. Knowles B. B. Solter D. (2014). DNA methylation dynamics during epigenetic reprogramming in the germline and preimplantation embryos. Genes Dev. 28, 812–828. 10.1101/gad.234294.113 24736841 PMC4003274

[B28] Morgan H. D. Sutherland H. G. Martin D. I. Whitelaw E. (1999). Epigenetic inheritance at the agouti locus in the mouse. Nat. Genet. 23, 314–318. 10.1038/15490 10545949

[B29] NCBI Resource Coordinators (2013). Database resources of the national center for Biotechnology information. Nucleic Acids Res. 41, D8–D20. 10.1093/nar/gks1189 23193264 PMC3531099

[B30] Ponsuksili S. Trakooljul N. Basavaraj S. Hadlich F. Murani E. Wimmers K. (2019). Epigenome-wide skeletal muscle DNA methylation profiles at the background of distinct metabolic types and ryanodine receptor variation in pigs. BMC Genomics 20, 492. 10.1186/s12864-019-5880-1 31195974 PMC6567458

[B31] Rakyan V. K. Chong S. Champ M. E. Cuthbert P. C. Morgan H. D. Luu K. V. (2003). Transgenerational inheritance of epigenetic states at the murine Axin(Fu) allele occurs after maternal and paternal transmission. Proc. Natl. Acad. Sci. U. S. A. 100, 2538–2543. 10.1073/pnas.0436776100 12601169 PMC151376

[B32] Rawlings N. Evans A. C. Chandolia R. K. Bagu E. T. (2008). Sexual maturation in the bull. Reprod. Domest. Anim. 43, 295–301. 10.1111/j.1439-0531.2008.01177.x 18638138

[B33] Schenkel F. S. (2021). Prospects for exploiting epigenetic effects in livestock production. Anim. Front. 11, 3–4. 10.1093/af/vfab071 34158984

[B34] Shoemaker R. Deng J. Wang W. Zhang K. (2010). Allele-specific methylation is prevalent and is contributed by CpG-SNPs in the human genome. Genome Res. 20, 883–889. 10.1101/gr.104695.109 20418490 PMC2892089

[B35] Tchurikov N. A. Kravatsky Y. V. (2021). The role of rDNA clusters in global epigenetic gene regulation. Front. Genet. 12, 730633. 10.3389/fgene.2021.730633 34531902 PMC8438155

[B36] Thompson P. J. Macfarlan T. S. Lorincz M. C. (2016). Long terminal repeats: from parasitic elements to building blocks of the transcriptional regulatory repertoire. Mol. Cell 62, 766–776. 10.1016/j.molcel.2016.03.029 27259207 PMC4910160

[B37] Triantaphyllopoulos K. A. Ikonomopoulos I. Bannister A. J. (2016). Epigenetics and inheritance of phenotype variation in livestock. Epigenetics Chromatin 9, 31. 10.1186/s13072-016-0081-5 27446239 PMC4955263

[B38] Wang M. Bissonnette N. Dudemaine P. L. Zhao X. Ibeagha-Awemu E. M. (2021). Whole genome DNA methylation variations in mammary gland tissues from Holstein cattle producing milk with various fat and protein contents. Genes (Basel) 12, 1727. 10.3390/genes12111727 34828333 PMC8618717

[B39] Xavier M. J. Roman S. D. Aitken R. J. Nixon B. (2019). Transgenerational inheritance: how impacts to the epigenetic and genetic information of parents affect offspring health. Hum. Reprod. Update 25, 518–540. 10.1093/humupd/dmz017 31374565

[B40] Zeng L. Wang M. Zhou J. Wang X. Zhang Y. Su P. (2022). A hypothesis: retrotransposons as a relay of epigenetic marks in intergenerational epigenetic inheritance. Gene 817, 146229. 10.1016/j.gene.2022.146229 35063571

[B41] Zeng Y. Chen T. (2019). DNA methylation reprogramming during mammalian development. Genes 10, 257. 10.3390/genes10040257 30934924 PMC6523607

[B42] Zhi D. Aslibekyan S. Irvin M. R. Claas S. A. Borecki I. B. Ordovas J. M. (2013). SNPs located at CpG sites modulate genome-epigenome interaction. Epigenetics 8, 802–806. 10.4161/epi.25501 23811543 PMC3883783

