# Supplementary material for: Defining bovine CpG epigenetic diversity by analyzing RRBS data from sperm of Montbéliarde and Holstein bulls
Source: Front Cell Dev Biol. 2025 Feb 20;13:1532711. doi: 10.3389/fcell.2025.1532711 (PMC11882585; doi:10.3389/fcell.2025.1532711)
Supplement: Supplementary file 2 [file Table1.docx]

**Supplementary Table S1.** Samples selected for CpG methylation call in bull sperm.

| **ID** | **Run** | **Breed** | **Age weeks** | **Bull** | **Treatment** | **Reads** |
| --- | --- | --- | --- | --- | --- | --- |
| ERX3767377 | ERR3765624 | Holstein | 69-71 | 51 | Low diet during early life | 28.7M |
| ERX3767379 | ERR3765626 | Holstein | 69-71 | 54 | Low diet during early life | 32.2M |
| ERX3767381 | ERR3765628 | Holstein | 69-71 | 59 | Low diet during early life | 26.5M |
| ERX3767383 | ERR3765630 | Holstein | 69-71 | 62 | Medium diet during early life | 26.5M |
| ERX3767385 | ERR3765632 | Holstein | 69-71 | 67 | Medium diet during early life | 33.6M |
| ERX3767387 | ERR3765634 | Holstein | 69-71 | 68 | Medium diet during early life | 25.5M |
| ERX3767389 | ERR3765636 | Holstein | 69-71 | 73 | High diet during early life | 26.1M |
| ERX3767391 | ERR3765638 | Holstein | 69-71 | 77 | High diet during early life | 34.3M |
| ERX3767393 | ERR3765640 | Holstein | 69-71 | 80 | High diet during early life | 34.5M |
| ERX6138345 | ERR6511509 | Montbéliarde | 74-82 | 1_4 | High Fertility | 32.1M |
| ERX6138348 | ERR6511512 | Montbéliarde | 74-82 | 1_7 | High Fertility | 35M |
| ERX6138354 | ERR6511518 | Montbéliarde | 74-82 | 11_13 | High Fertility | 32.6M |
| ERX6138364 | ERR6511528 | Montbéliarde | 74-82 | 2_12 | High Fertility | 35.3M |
| ERX6138381 | ERR6511545 | Montbéliarde | 74-82 | 2_6 | High Fertility | 38.7M |
| ERX6138401 | ERR6511565 | Montbéliarde | 74-82 | 3_24 | High Fertility | 27.3M |
| ERX6138410 | ERR6511574 | Montbéliarde | 74-82 | 5_21 | High Fertility | 35.8M |
| ERX6138420 | ERR6511584 | Montbéliarde | 74-82 | 6_15 | High Fertility | 34.1M |
| ERX6138435 | ERR6511599 | Montbéliarde | 74-82 | 6_8 | High Fertility | 40.7M |
