# Supplementary material for: Defining bovine CpG epigenetic diversity by analyzing RRBS data from sperm of Montbéliarde and Holstein bulls
Source: Front Cell Dev Biol. 2025 Feb 20;13:1532711. doi: 10.3389/fcell.2025.1532711 (PMC11882585; doi:10.3389/fcell.2025.1532711)
Supplement: Supplementary file 3 [file Table8.docx]

**Supplementary Table S8.** Percentage of overlapping with repeat elements for DMCs or random SfCs subsets.

| Feature | DMC | Subset 1 | Subset 2 | Subset 3 | Subset 4 | Subset 5 | Subset 6 | Subset 7 | Subset 8 | Subset 9 | Subset 10 | Subset Av | Enrichment |
| --- | --- | --- | --- | --- | --- | --- | --- | --- | --- | --- | --- | --- | --- |
| (GCGT)n#Simple_repeat | 11 | 0 | 0 | 0 | 0 | 0 | 1 | 0 | 0 | 0 | 0 | 0.1 | 110.0 |
| 5S#rRNA | 12 | 0 | 1 | 0 | 1 | 0 | 0 | 2 | 0 | 0 | 1 | 0.5 | 24.0 |
| BOV-A2#SINE/Core-RTE | 65 | 19 | 20 | 18 | 12 | 17 | 19 | 19 | 17 | 25 | 19 | 18.5 | 3.5 |
| BovB#LINE/RTE-BovB | 23 | 21 | 14 | 21 | 17 | 18 | 23 | 17 | 27 | 20 | 15 | 19.3 | 1.2 |
| Bov-tA1#SINE/tRNA-Core-RTE | 88 | 39 | 51 | 46 | 51 | 56 | 52 | 53 | 41 | 51 | 51 | 49.1 | 1.8 |
| Bov-tA2#SINE/tRNA-Core-RTE | 59 | 35 | 25 | 34 | 28 | 36 | 26 | 30 | 26 | 39 | 38 | 31.7 | 1.9 |
| Bov-tA3#SINE/tRNA-Core-RTE | 110 | 59 | 55 | 59 | 45 | 51 | 41 | 67 | 49 | 58 | 45 | 52.9 | 2.1 |
| BTSAT2#Satellite/centr | 42 | 3 | 1 | 6 | 7 | 5 | 4 | 6 | 7 | 6 | 3 | 4.8 | 8.8 |
| BTSAT4#Satellite/centr | 181 | 10 | 13 | 25 | 23 | 16 | 19 | 14 | 17 | 23 | 13 | 17.3 | 10.5 |
| CHR-2#SINE/tRNA | 25 | 8 | 18 | 10 | 14 | 13 | 12 | 12 | 10 | 11 | 14 | 12.2 | 2.0 |
| CHR-2A#SINE/tRNA | 85 | 25 | 21 | 35 | 20 | 33 | 23 | 15 | 20 | 26 | 27 | 24.5 | 3.5 |
| CHR-2B#SINE/tRNA | 31 | 21 | 18 | 14 | 11 | 13 | 15 | 15 | 21 | 15 | 22 | 16.5 | 1.9 |
| CHRL#SINE/tRNA | 12 | 15 | 5 | 8 | 9 | 10 | 7 | 7 | 8 | 7 | 14 | 9 | 1.3 |
| ERV2-1-LTR_BT#LTR/ERVK | 63 | 3 | 5 | 5 | 4 | 4 | 6 | 4 |  | 3 | 5 | 4.3 | 14.5 |
| L1_BT#LINE/L1 | 10 | 3 | 2 | 1 | 3 | 2 | 2 | 4 | 4 | 5 | 1 | 2.7 | 3.7 |
| L1-2_BT#LINE/L1 | 36 | 12 | 8 | 10 | 5 | 7 | 13 | 12 | 7 | 9 | 5 | 8.8 | 4.1 |
| L1MC4#LINE/L1 | 9 | 0 | 2 | 0 | 1 | 1 | 3 | 2 | 2 | 1 | 1 | 1.3 | 6.9 |
| L2a#LINE/L2 | 27 | 21 | 21 | 15 | 28 | 17 | 18 | 24 | 23 | 9 | 19 | 19.5 | 1.4 |
| L2b#LINE/L2 | 15 | 15 | 20 | 10 | 15 | 12 | 17 | 7 | 16 | 17 | 16 | 14.5 | 1.0 |
| L2c#LINE/L2 | 8 | 7 | 3 | 14 | 9 | 8 | 8 | 5 | 9 | 11 | 7 | 8.1 | 1.0 |
| L2d2#LINE/L2 | 12 | 5 | 7 | 2 | 1 | 3 | 3 | 1 | 4 | 2 | 2 | 3 | 4.0 |
| LSU-rRNA_Hsa#rRNA | 18 | 0 | 0 | 1 | 0 | 2 | 0 | 0 | 0 | 1 | 0 | 0.4 | 45.0 |
| LTR16A#LTR/ERVL | 10 | 1 | 2 | 0 | 2 | 0 | 1 | 1 | 0 | 0 | 1 | 0.8 | 12.5 |
| MIR#SINE/MIR | 10 | 6 | 14 | 17 | 10 | 13 | 13 | 13 | 8 | 11 | 15 | 12 | 0.8 |
| MIRb#SINE/MIR | 23 | 14 | 14 | 17 | 23 | 12 | 16 | 20 | 11 | 22 | 14 | 16.3 | 1.4 |
| MIRc#SINE/MIR | 12 | 6 | 7 | 7 | 5 | 5 | 11 | 9 | 6 | 8 | 6 | 7 | 1.7 |
