# Supplementary material for: Defining bovine CpG epigenetic diversity by analyzing RRBS data from sperm of Montbéliarde and Holstein bulls
Source: Front Cell Dev Biol. 2025 Feb 20;13:1532711. doi: 10.3389/fcell.2025.1532711 (PMC11882585; doi:10.3389/fcell.2025.1532711)
Supplement: Supplementary file 4 [file Table7.docx]

**Supplementary Table S7.** Number of cytosines that are adjacent (1bp) close (2000 bp) or distant (>2000bp) in DMCs and in the ten Subsets. P Value was calculated with t-Test.

| **Distance between adjacent cytosines** | **1bp** | **2bp-2Kb** | **>2Kb** |
| --- | --- | --- | --- |
| **near DMCs** | 478 | 2149 | 3447 |
| **near r-Cs Subset1** | 14 | 609 | 5451 |
| **near r-Cs Subset2** | 14 | 581 | 5479 |
| **near r-Cs Subset3** | 12 | 582 | 5480 |
| **near r-Cs Subset4** | 12 | 594 | 5468 |
| **near r-Cs Subset5** | 12 | 632 | 5430 |
| **near r-Cs Subset6** | 17 | 600 | 5457 |
| **near r-Cs Subset7** | 16 | 602 | 5456 |
| **near r-Cs Subset8** | 19 | 600 | 5455 |
| **near r-Cs Subset9** | 19 | 602 | 5453 |
| **near r-Cs Subset10** | 18 | 607 | 5449 |
| **near r-Cs Subset Average** | 15.3 | 600.9 | 5458 |
| **Enrichment near DMCs/near SubsetsAv** | 31.2 | 3.6 | 0.6 |
| **Pvalue** | < 1.0e-15 | < 1.0e-15 | < 1.0e-15 |
