# Supplementary material for: Defining bovine CpG epigenetic diversity by analyzing RRBS data from sperm of Montbéliarde and Holstein bulls
Source: Front Cell Dev Biol. 2025 Feb 20;13:1532711. doi: 10.3389/fcell.2025.1532711 (PMC11882585; doi:10.3389/fcell.2025.1532711)
Supplement: Supplementary file 5 [file Table2.docx]

**Supplementary Table S2.** Samples selected for SNP detection in Holstein and Montbéliarde breeds.

| **ID** | **Run** | **Breed** | **Sample Name** | **Reads** | **Gb** |
| --- | --- | --- | --- | --- | --- |
| ERX963066 | ERR883663 | Holstein | INRA_HOL0004 | 233.0 | 47.1 |
| ERX963068 | ERR883665 | Holstein | INRA_HOL0006 | 154.2 | 31.1 |
| ERX963071 | ERR883668 | Holstein | INRA_HOL0009 | 149.9 | 30.3 |
| ERX963078 | ERR883675 | Holstein | INRA_HOL0016 | 161.8 | 32.4 |
| ERX963083 | ERR883680 | Holstein | INRA_HOL0021 | 173.3 | 35.0 |
| ERX963091 | ERR883688 | Montbéliarde | INRA_MON0002 | 128.2M | 25.9 |
| ERX963094 | ERR883691 | Montbéliarde | INRA_MON0005 | 137.8 | 27.8 |
| ERX963095 | ERR883692 | Montbéliarde | INRA_MON0006 | 251.0M | 50.7 |
| ERX963098 | ERR883695 | Montbéliarde | INRA_MON0009 | 134.2M | 27.1 |
| ERR883696 | ERR883696 | Montbéliarde | INRA_MON0010 | 106.0M | 21.4 |
