# Supplementary material for: Defining bovine CpG epigenetic diversity by analyzing RRBS data from sperm of Montbéliarde and Holstein bulls
Source: Front Cell Dev Biol. 2025 Feb 20;13:1532711. doi: 10.3389/fcell.2025.1532711 (PMC11882585; doi:10.3389/fcell.2025.1532711)
Supplement: Supplementary file 6 [file DataSheet2.docx]

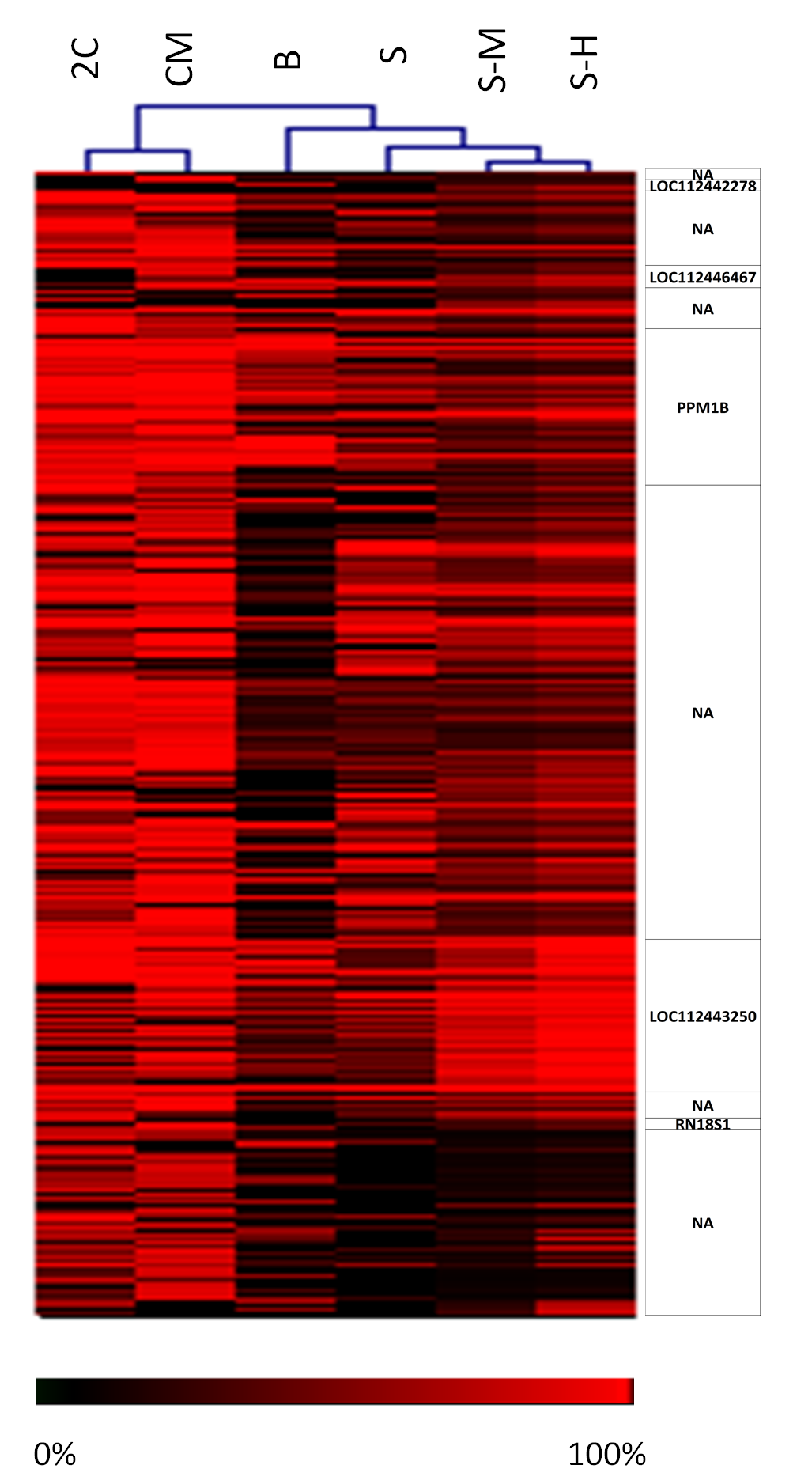


**Supplementary Figure S2**. Hierarchical Clustering Analysis of methylation level of a subset of 308 differentially methylated cytosines (DMCs) in sperm from Holstein (S-H) and Montbéliarde (S-M) breeds that found correspondence with RRBS data from cells isolated in different stages of cattle embryogenesis: 2-Cell (2C), Compact Morula (CM), Blastocyst (B) and Spermatozoa (S) (Jiang et al., 2018). When present, genes close to DMCs (2Kb) were reported (NA = Not Annotated).
