# Supplementary material for: Defining bovine CpG epigenetic diversity by analyzing RRBS data from sperm of Montbéliarde and Holstein bulls
Source: Front Cell Dev Biol. 2025 Feb 20;13:1532711. doi: 10.3389/fcell.2025.1532711 (PMC11882585; doi:10.3389/fcell.2025.1532711)
Supplement: Supplementary file 7 [file Table4.docx]

**Supplementary Table S4.** Incidence of SNPs in CpG positions for DMCs, r-Cs random subsets and total Cs w/o DMCs.

|  |  | **2bp** | **10bp** | **100bp** | **2kb** |
| --- | --- | --- | --- | --- | --- |
| **DMCs (n=6,074)** | SNPs | 51 | 117 | 533 | 2759 |
|  | SNPs/DMCs % | 0.84 | 1.93 | 8.78 | 45.42 |
| **r-Cs Average 10 subsets (n=6,074)** | SNPs | 1.78 | 9.60 | 91.10 | 2028.00 |
|  | SNPs/DMCs % | 0.03 | 0.16 | 1.50 | 33.39 |
| **Total r-Cs (n=350,561)** | SNPs | 86 | 541 | 5260 | 116783 |
|  | SNPs/DMCs % | 0.02 | 0.15 | 1.50 | 33.30 |
