# Supplementary material for: Defining bovine CpG epigenetic diversity by analyzing RRBS data from sperm of Montbéliarde and Holstein bulls
Source: Front Cell Dev Biol. 2025 Feb 20;13:1532711. doi: 10.3389/fcell.2025.1532711 (PMC11882585; doi:10.3389/fcell.2025.1532711)
Supplement: Supplementary file 8 [file Table6.docx]

**Supplementary Table S6.** H vs M cytosine positions compared to tau_ind_DMC positions (Capra et al., 2023) for DMCs (n=6,074), 10 different r-Cs random subsets (n=6,074 each) and total r-Cs. Matches are considered according to distance of H vs M cytosines from tau_ind_DMCs: 2Kbp=less than 2Kbp, 100=less than 100bp, 10bp=less than 10bp, 2bp=less than 2bp.

|  |  | **2bp** | **10bp** | **100bp** | **2kb** |
| --- | --- | --- | --- | --- | --- |
| **DMCs (n=6,074)** | tau_ind DMCs | 74 | 110 | 367 | 1526 |
|  | tau_ind DMCs/DMCs % | 1.2 | 1.8 | 6 | 25.1 |
| **r-Cs Average 10 subsets (n=6,074)** | tau_ind DMCs | 13.4 | 19 | 96.7 | 842.2 |
|  | tau_ind DMCs/DMCs % | 0.2 | 0.3 | 1.6 | 13.9 |
| **Total r-Cs (n=350,561)** | SNPs | 746 | 1080 | 5632 | 48854 |
|  | SNPs/DMCs % | 0.2 | 0.3 | 1.6 | 13.9 |
