# Supplementary material for: Defining bovine CpG epigenetic diversity by analyzing RRBS data from sperm of Montbéliarde and Holstein bulls
Source: Front Cell Dev Biol. 2025 Feb 20;13:1532711. doi: 10.3389/fcell.2025.1532711 (PMC11882585; doi:10.3389/fcell.2025.1532711)
Supplement: Supplementary file 10 [file DataSheet1.docx]

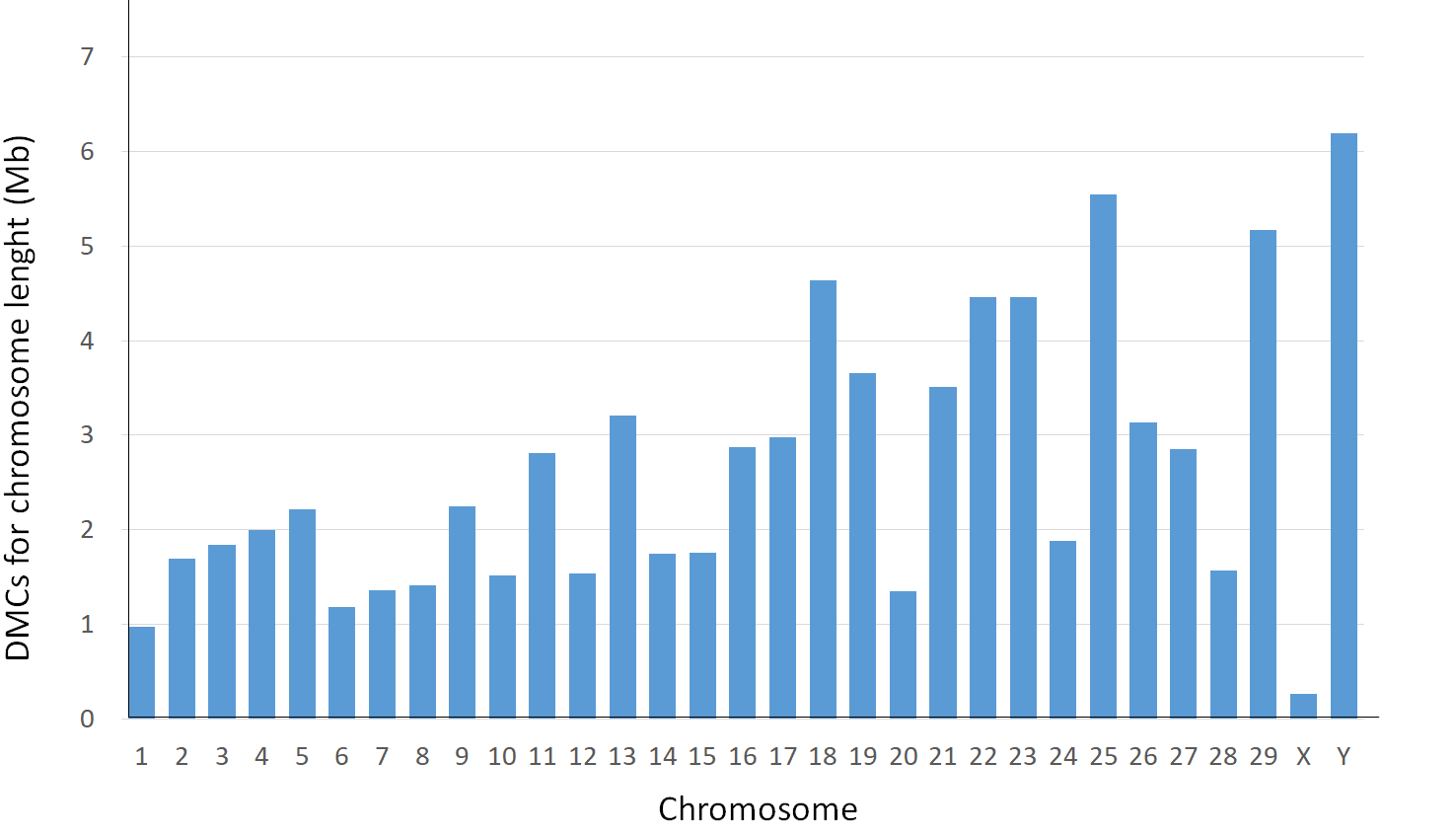


**Supplementary Figure S1.** Number of differentially methylated cytosines (DMCs) between the two breeds normalized for the lenght of each chromosome in megabases (Mb).
